# The Value of *In Vitro* Diagnostic Testing in Medical Practice: A Status Report

**DOI:** 10.1371/journal.pone.0149856

**Published:** 2016-03-04

**Authors:** Ulrich-Peter Rohr, Carmen Binder, Thomas Dieterle, Francesco Giusti, Carlo Guiseppe Mario Messina, Eduard Toerien, Holger Moch, Hans Hendrik Schäfer

**Affiliations:** 1 Roche Diagnostics, Sequencing Unit, Pleasanton, CA, 94588, United States of America; 2 Institute of Surgical Pathology, University Hospital Zurich, 8091, Zurich, Switzerland; 3 Kantonsspital Baselland, 4410, Liestal, Switzerland; 4 European Organisation for Research and Treatment of Cancer, 1200, Brussels, Belgium; 5 Divisional Medical and Scientific Affairs, F. Hoffmann-La Roche Ltd, 4070, Basel, Switzerland; 6 Faculty of Economic and Management Sciences, University of Pretoria, 0060, Pretoria, South Africa; 7 Institute of Anatomy II, University Hospital Jena, Friedrich Schiller University, 07740, Jena, Germany; National Institute for Viral Disease Control and Prevention, CDC, China, CHINA

## Abstract

**Background:**

*In vitro* diagnostic (IVD) investigations are indispensable for routine patient management. Appropriate testing allows early-stage interventions, reducing late-stage healthcare expenditure (HCE).

**Aim:**

To investigate HCE on IVDs in two developed markets and to assess the perceived value of IVDs on clinical decision-making. Physician-perceived HCE on IVD was evaluated, as well as desired features of new diagnostic markers.

**Methods:**

Past and current HCE on IVD was calculated for the US and Germany. A total of 79 US/German oncologists and cardiologists were interviewed to assess the number of cases where: physicians ask for IVDs; IVDs are used for initial diagnosis, treatment monitoring, or post-treatment; and decision-making is based on an IVD test result. A sample of 201 US and German oncologists and cardiologists was questioned regarding the proportion of HCE they believed to be attributable to IVD testing. After disclosing the actual IVD HCE, the physician’s perception of the appropriateness of the amount was captured. Finally, the association between physician-rated impact of IVD on decision-making and perceived contribution of IVD expenditure on overall HCE was assessed.

**Results:**

IVD costs account for 2.3% and 1.4% of total HCE in the US and Germany. Most physicians (81%) believed that the actual HCE on IVDs was >5%; 19% rated the spending correctly (0–4%, p<0.001). When informed of the actual amount, 64% of physicians rated this as appropriate (p<0.0001); 66% of decision-making was based on IVD. Significantly, more physicians asked for either additional clinical or combined clinical/health economic data than for the product (test/platform) alone (p<0.0001).

**Conclusions:**

Our results indicate a poor awareness of actual HCE on IVD, but a high attributable value of diagnostic procedures for patient management. New markers should deliver actionable and medically relevant information, to guide decision-making and foster improved patient outcomes.

## Introduction

In vitro diagnostic (IVD) testing has become an indispensable tool in clinical practice for diagnosing and monitoring of diseases, as well as providing prognosis and predicting treatment response [1, 2]. In addition, IVD is used to assess the potential risk of developing a disease or disorder and to guide patient management [1]. IVD of analytes originating from body specimens, including blood and tissue biopsies, is used alone or in combination with clinical investigations [2] and is perceived as an important tool for high-quality medical outcomes [3]. There are over 40,000 different IVD products available that provide information to doctors and patients on a huge range of conditions. These comprise markers for inorganic chemistry (electrolytes, toxins, and heavy metals), markers for organic chemistry/biochemistry (proteins, lipids, and carbohydrates), as well as molecular biologic procedures (sequencing and polymerase chain reaction). One German study revealed that up to 187 of 584 diagnoses can be confirmed exclusively by an IVD testing [4]. Routine diagnostics and population screening programs, such as the Pap smear for cervical carcinoma, have the potential to identify high-risk individuals and to prevent disease onset or progression [5, 6]. The introduction of cervical cancer screening programs in Europe has led to a substantial decrease in mortality [7, 5]. Furthermore, timely IVD testing allows more early-stage and cost-effective interventions, instead of advanced-stage therapy, which is generally associated with worse prognosis and a higher use of healthcare resources [8, 9].

### A New Trend towards Companion Diagnostics

The contemporary concept of companion diagnostics is based on identifying patients with a high likelihood of response to a specific drug, hence curbing total costs of healthcare due to targeted patient management. A well-known example is Herceptest^®^–the companion diagnostic for HER2-positive breast cancer and gastric cancer–which identifies patients eligible for trastuzumab treatment [10]. Other examples of Food and Drug Administration (FDA)-approved drugs with companion diagnostics include cetuximab, imatinib, and vemurafenib, which are used to treat metastatic colorectal cancer, gastrointestinal stroma tumor, and late-stage melanoma, respectively [11, 12] With the emergence of new molecular technologies identifying tumor aberrations that can be treated with targeted agents, the number of companion diagnostic tests used in oncology will significantly increase in the future.

Companion diagnostics has the potential to enable the selection of the correct drug dose at the appropriate time of a patient`s treatment course, thereby reducing overall therapy cost. The investment of developing companion diagnostic drugs is substantial, however [13]. This is particularly true for immunotherapy treatments, such as those targeting cytotoxic T-lymphocyte antigen 4 (CTLA-4), programmed cell death-1 (PD-1), or programmed cell death 1 ligand 1 (PD-L1). For example, the cost of using the PD-1 inhibitor pembrolizumab has already exceeded an annual cost per patient of US$1 million [13]. In an initiative to help manage costs while maintaining high quality care, the American Society of Clinical Oncology (ASCO) has recently proposed a framework to assess the value of cancer treatment options [14].

### Value in Healthcare

The question of how to measure value in healthcare has been discussed controversially. Nonetheless, there is agreement on the overarching concept of assessing health outcomes achieved per dollar spent [15]. In economic terms, the value component would equal clinical utility and cost-effectiveness [16]. The value term simultaneously involves patients (utility and efficacy) and payers (efficiency), describing a framework for performance improvement in healthcare [17]. The Joint Commission of Healthcare Organizations has defined the value term as “the degree to which patient care services increase the probability of desired patient outcomes and reduce the probability of undesired outcomes, given the current state of knowledge” [18].

### The Value of IVD

IVD tests have been under increasing cost pressure over the last decade as a result of their increasing use and concerns about uncontrollable healthcare expenditures [19]. Furthermore, the diagnostic industry is now facing stricter regulatory hurdles for product approval [20]. For many years, registration of diagnostic tests in the European Union only required the CE label; however, in the light of financial shortfalls, health authorities are increasingly requesting proof that diagnostic tests not only have reasonable pricing, but also add considerable value to society [21].

Many articles introduce frameworks of how best to assess the value of laboratory diagnostics [22, 23]. Basically, IVD value may be defined as:

IVD value=Performance×Efficiency

IVD value=[Technical accuracy/Turnaround time]×[Utility/Costs]

Performance is mandated to give the highest accuracy, referring to outcome reliability and reproducibility, with the lowest turnaround time. Efficiency is derived from the percentage of confident clinical decisions made (clinical utility) over costs. While costs refer to resource usage for a given process [24], utility speaks to driving the most accurate conclusion given available evidence for a diagnostic test [25].

While the performance of IVD testing devices is fairly comparable across the diagnostic industry, the efficiency component is the main differentiator and determines the medical value component. Although the utility–cost relationship is difficult to assess, it is important to quantify this amount and to estimate the current value of IVD testing in proportion to its cost relative to overall healthcare expenditure (HCE).

#### Aims

In 2005, a report by the Lewin Group revealed that diagnostics comprise less than 5% of hospital costs and approximately 1.6% of all Medicare costs, while accounting for 60–70% of clinical decisions [26]. However, the authors do not provide citable references for their claims. The main objective of this study was to find statistical backing for the surprising utility–cost ratio.

## Methods

This study was conducted in three stages: literature review, interviews with medical oncologists and cardiologists, and a confirmatory internet-based multiple-choice survey (SERMO). Given that the study was based on interview responses and did not involve active treatment of human participants, it was not necessary to include an institutional review board (ethics committee).

Table 1 provides an overview of all stages of the study.

**Table 1:** Study Design.

Study Stage	Quality	Objectives/Aims	Methods	Countries	Physicians
**Stage 1**	Quantitative	• % of healthcare expenditures used for IVD	Systematic literature research	Germany and US	None
		• % of healthcare expenditures used for IVD in hospital and private practice			
**Stage 2**	Qualitative & Quantitative	• Patients seen per week	Interviews	Germany	Onc (N = 20) Card (N = 20
		• Distribution		US	Onc (N = 20)
		○ New patients			Card (N = 19)
		○ Patients undergoing treatment			
		○ Patients in post-treatment phase			
		• Overall and specific amount of IVD testing			
		○ In initial diagnostic phase			
		• IVD subtype use			
		• Rated importance of IVD subtype			
		○ In treatment phase			
		○ In post-treatment follow-up			
		• Treatment decision based on IVD-testing			
**Stage 3**	Quantitative	• % of healthcare expenditures used for IVD	Questionnaire	Germany	Onc (N = 30) Card (N = 51)
		• Perceived HCE on IVD testing		US	Onc (N = 70) Card (N = 50)
		• Perception of spending appropriateness			
		• Design of optimal biomarker		Germany, US, UK, Canada, Norway, Switzerland	Onc (N = 102) Card (N = 102) GP (N = 38) Int. M (N = 38) Path (N = 68)

Display of study design, objectives and methods used in the three different parts of the analysis. Number of sources, included physicians, their specializations and country of origin.

IVD, *in-vitro* diagnostic; Onc, oncologist; Card, cardiologist; GP, general practitioner; Int. M, internal medicine; Path, pathologist; HCE, healthcare expenditure.

### Stage 1

A literature review was conducted to assess the total percentage of HCE on IVDs in two countries–the US and Germany. Data for healthcare and diagnostic expenditures were derived from government and private industry sources. Secondary sources were assessed for information and data on IVD HCE including government websites, healthcare agencies, industry, and market reports. Various search terms were used to ascertain IVD spending data, including “clinical laboratory industry revenues”, “in-vitro diagnostic spending”, “clinical laboratory market”, and “reimbursement for clinical laboratories”.

Data for total US HCE for the period 1993–2011 were derived from the Centers for Medicare and Medicaid Services [27]. At the time of this analysis, 2009–2013 data for IVD expenditure in the US had not yet been published, therefore an estimate for spending during that period was made based on an average annual growth rate of 4% [28]. For the years 1994–1997, an average growth rate of 5.3% was applied. The 1993 IVD spending was an estimate based on the ratio of IVD manufacturers’ revenues over total IVD spending in other years.

For Germany, both total government healthcare spending and IVD spending were captured from the Federal Statistical Office [29]. All calculations were based on German government HCE, which accounts for about 77% of overall healthcare costs [30].

The percentage of total healthcare spending on IVDs was calculated by dividing the total IVD spending by the total HCE.

#### Selection of Countries

**Fig 1:**
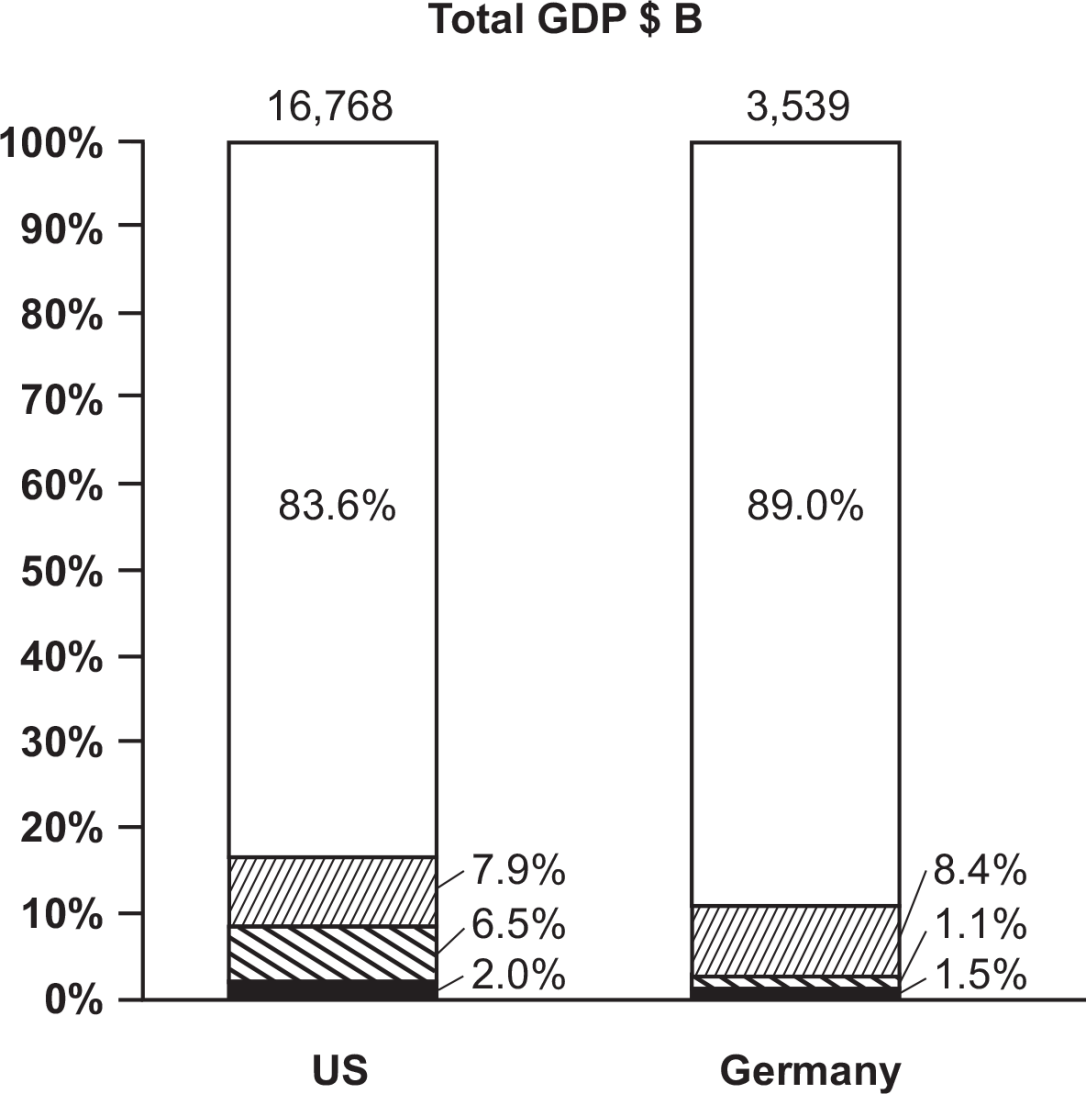
Percentage of public and private HCE of GDP (pie) in the US and Germany in 2013. Thin lines, public HCE; bold lines, private HCE without out-of-pocket; black fill, out-of-pocket HCE; white fill, rest of GDP (non-HCE) HCE, healthcare expenditure; GDP, gross domestic product; $B, US$ billion.

The US and Germany were selected as they represented approximately the estimated global HCE in 2009 [31] and therefore provide satisfactory proxies for other countries in developed markets. Fig 1 displays the percentage of GDP allocated to HCE and the total HCE split according to payers for both countries in 2013.

### Stage 2

Interviews were conducted with medical oncologists and cardiologists in the US and Germany. The interview was designed to assess (i) the number of patients seen per week and (ii) the distribution of patients according to treatment stages. Furthermore we assessed (iii) in how many cases physicians were asking for IVD and (iv) in how many cases IVD was used for either initial diagnosis, treatment monitoring or post-treatment follow up. With this in mind we also analyzed which IVD subtypes were used frequently during initial patient work up and how important these subtypes were rated by physicians. We then investigated (v) in how many cases a treatment decision (defined as stopping, initiating or continuing treatment) is based on IVD-test results.

Interviews were conducted by three neutral researchers (all male; one MD, one PhD, and one BS, MBA) employed by the Enterprise Analysis Corporation (EAC; Stamford, CT, US). Interviewers had a strong knowledge of diagnostics testing and were experienced in conducting interviews with physicians, laboratory workers and other healthcare professionals.

Participants were contacted by telephone and asked to participate in the study; the nature of the study was briefly described and an honorarium was offered for participation. Interviewers had no prior relationship with physicians. Physicians were required to see ≥20 patients per week in their practice. These interviews took place by phone and were scheduled to take 1 hour; no non-participants were involved in the interviews. A pilot-tested, structured interview was conducted. Interview questions were not shared with the physician in advance of the interview. There were no repeat interviews. In general, interviews were not recorded although some may have been if the physician consented to recording. Notes were taken during the interview; additional comments and notes were added to the interview protocol directly after the interview whilst fresh in the mind of the interviewer. Transcripts were not returned as there were few open-ended questions and transcripts were not lengthy.

Data from the interviews were entered into a database (Microsoft Access) by one person; this was reviewed for accuracy by a project manager at EAC.

### Stage 3

This stage involved a confirmatory internet-based multiple-choice survey of physician, the purpose of which was (vi) to assess how much physicians believed to be spent on IVD testing and to compare their assumption with the actual HCE spent on IVD calculated in Stage 1 and (vii) to assess, after disclosing the actual HCE on IVDs, if physicians felt this amount was appropriate. Finally, the perceived value of IVDs was correlated with the physician’s estimated cost.

Stage 3 was executed using SERMO, an anonymized internet-based multiple-choice survey, executed by Genentech (South San Francisco, CA, US), hosted by WorldOne (Boston, MA, US). SERMO is a shared service, with a facility that allows multiple companies to gain quick and comprehensive insights on conceptual questions via physician surveys. Interested physicians apply to take part in surveys and receive questions from several companies. Invitations to participate in this cross-sectional survey were sent to physicians from the US, UK, Germany, Canada, Norway, and Switzerland; 348 physicians responded to the invitation to participate. Participating physicians received financial compensation.

### Statistical Analysis

Student’s *t* test was applied to compare mean values of patient numbers seen by physicians, by country and specialty (Germany vs US; cardiologists vs oncologists). A comparison of patients undergoing IVD testing between countries and specialties was performed with a χ^2^ test (with 3 degrees of freedom) after recalculating the number of patients by weighted average. The correlation between the replies regarding value of IVD in clinical practice and perceived costs was estimated with the Spearman’s rank correlation coefficient. A Likert Scale (1 = lowest importance, 5 = highest importance) was used for the assessment of the relative importance of IVD subtypes during the initial patient work up phase. All statistical analyses were performed using SAS version 9.4 (SAS Institute Inc, Cary, NC, US).

## Results

### Stage 1

#### IVD Spending as a Percentage of Total HCE

**Fig 2:**
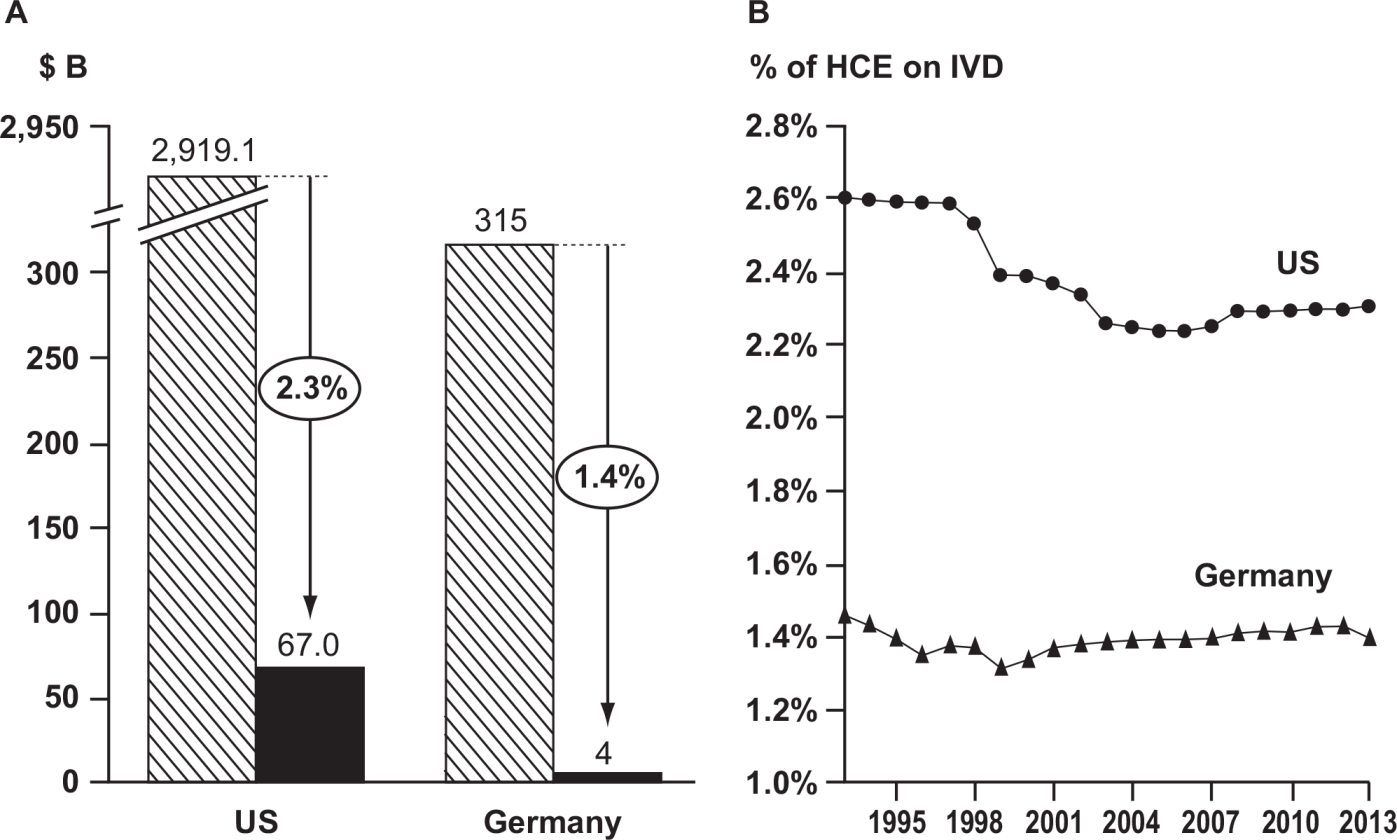
**(A) Percentage of HCE on IVD in 2013 and (B) evolution of HCE on IVD 1993–2013.** HCE, healthcare expenditure; IVD, *in vitro* diagnostics; $ B, US$ billion.

The literature research revealed that healthcare spending on IVDs (defined as payments to clinical laboratories for testing services) represents approximately 2.3% of all healthcare spending in the US (S1 Table). In Germany, 1.4% of public healthcare expenditure is used for IVD (S1 Table; Fig 2A). Although government spending on IVD testing is well documented, private sector spending is not systematically tracked on an annual basis, thus fewer data resources are available.

A retrospective analysis from 1993 through 2013 revealed that IVD spending in the US has grown at an annual rate of 5.3% from US$30 billion in 1998 to an estimated US$67 billion in 2013. In Germany, spending on IVD has grown at a more modest annual rate of 3.1% since 1993, reaching US$ 4.5 billion in 2013. Overall, this has resulted in a relatively consistent HCE on IVD testing in Germany, whereas a slight decline of 0.2% was observed in the US (Fig 2B).

### Stage 2

#### IVD Use for Patients and Clinical Decision-Making

A total of 40 oncologists and 39 cardiologists participated in physician interviews. On average, 93 patients were seen per week by oncologists and cardiologists in the US and Germany. Significantly more patients were treated by physicians in the US compared with Germany (p = 0.005). A comparable number of patients underwent IVD testing in the US (74%) and Germany (76%) (p = 0.119; average 75%). Overall, IVD testing was used in 88%, 77%, and 72% of patients for initial diagnosis, treatment monitoring, and follow-up, respectively.

Significantly more oncology patients underwent IVD testing than cardiology patients (92% vs 60%, respectively; p<0.0001) in both, the US (p<0.0001) and Germany (p<0.0001) (Table 2). Overall, 75% of patients underwent IVD testing across both disciplines, testing that led to a substantial clinical decision in 66% of these patients (Table 2).

**Table 2:** Number of Patients Receiving IVD Testing per Week and General Use of IVD Testing During Different Phases of Care.

			Patients treated with IVD			General use of IVD (%)		
Country	Specialty	Setting	Mean No. of patients/week	Patients with IVD testing (%)	Clinical decisions affected by IVD (%)	Initial diagnosis	Treatment monitoring	Post-treatment monitoring
**US**								
	Oncologist	**Total**	62	91	58	95	95	79
		Private	87	94	62	97	97	81
		Hospital	45	88	54	92	92	76
	Cardiologist	**Total**	86	62	68	86	52^a^	52^a^
		Private	99	42	59	79	44^a^	44^a^
		Hospital	74	80	76	93	61^a^	61^a^
	**Average US**		74	74	64	90	71	64
**Germany**								
	Oncologist	**Total**	114	92	63	100	94	87
		Private	95	88	58	99	92	77
		Hospital	133	96	67	100	96	95
	Cardiologist	**Total**	112	59	71	75	68^a^	68^a^
		Private	175	35	64	57	51^a^	51^a^
		Hospital	95	92	80	99	90^a^	90^a^
	**Average Germany**		113	76	67	87	81	78
**Overall**								
**Average oncologists**			88	92	62	97	94	84
**Average cardiologists**			99	60	70	82	61^a^	61^a^
**Average Overall (%)**				**75**	**66**	**88**	**77**	**72**
**Average Overall (in No. of patients/week)**			**93**	**71**	**61**			

IVD, *in vitro* diagnostics. ^a^For cardiologists, only one question was asked on treatment monitoring and post-treatment monitoring.

### IVD Subtype Use and Rated Subtype Importance

During the initial work-up phase, on average physicians used clinical chemistry and hematology assessments in nearly 100% of patients, followed by immunology (86%). Basic and Advanced Tissue Staining as well as molecular diagnostics was used in approximately half of patients during the initial work-up phase. The average rating of IVD subtype importance corresponded with its usage (S1 Fig). Detailed information about country- and specialty-related IVD usage and rated importance is displayed in S2 Table.

### Stage 3

#### Actual and Perceived Spending of IVD on HCE

Our analysis yielded a discrepancy between the actual amount of IVD on HCE (2.3% in the US; 1.4% in Germany) and the perceived amount of HCE by physicians. Throughout all specializations and irrespective of country, physicians tended to overestimate IVD-related monetary expenditure; this was most pronounced among US cardiologists. In total, 81% of physicians estimated IVD expenditure to be >5% of the total HCE.19% rated spending to be 0–4% (p<0.001) (Table 3).

**Table 3:** Physicians’ estimation of IVD expenditure as a proportion of total HCE.

	IVD expenditure, % of total HCE			
Country	0–4%	5–10%	11–20%	>20%
**Germany, % physicians**				
Oncologist	20.0	43.3	20.0	16.7
Cardiologist	17.6	49.0	25.5	7.8
Total	18.5	46.9	23.5	11.1
**US, % physicians**				
Oncologist	22.5	31.0	32.4	14.1
Cardiologist	14.0	38.0	26.0	22.0
Total	19.0	33.9	29.8	17.4
**Overall**	**18.8**	**39.1**	**27.2**	**14.9**

HCE, healthcare expenditure; IVD, *in vitro* diagnostic.

#### Assessment of Appropriateness of IVD Costs on Overall HCE

After disclosure of the actual proportion of HCE incurred by IVD testing (2.3% and 1.4% for the US and Germany, respectively), 92% of all physicians rated the current IVD expenditure as either appropriate or too low and 7% as too high (p<0.0001). No major differences were observed within countries (Spearman’s correlation coefficient 0.291 for Germany, 0.003 for US) or specialization (Spearman’s correlation coefficient 0.118 for cardiology, 0.031 for oncology).

On a more granular level, 64% of physicians rated current IVD spending as appropriate; 28% of physicians assessed this spending as too low. 8% of the latter believed that innovative assays deserve to command a higher price, even if total healthcare spend were to increase, while 20% of the latter that innovative assays deserve a higher price but cost cuts would need to be made in other segments of the healthcare value chain. Among the remaining 8% who rated the current spending as too high, 5% believed that IVD savings should be added to other segments of the healthcare value chain.

#### Association Between Impact of IVD on Clinical Decisions and Perceived Cost Contribution

Physicians who rated the impact of IVD testing as rather low also believed that the associated cost of such procedures was low. Conversely, physicians who rated the value of IVD testing as high considered it to be more expensive (Spearman’s rank correlation coefficient 0.28617; p≤0.0001). For example, 26.7% of physicians who based >85% of their clinical decisions on IVDs estimated the cost impact of IVDs at over 20% of the overall HCE (Table 4).

**Table 4:** Relationship between percentage of clinical decisions based on IVD testing and perceived HCE on IVDs by physicians.

Clinical decisions based on IVD	Perceived HCE on IVD			
	0–4%	5–10%	11–20%	>20%
**<20%**	34.7%***	40.8%***	16.3%**	8.2%*
**20–44%**	19.7%**	44.3%***	19.7%**	16.4%**
**45–64%**	7.7%*	36.5%***	40.4%***	15.4%**
**65–85%**	8.3%*	41.7%***	33.3%***	16.7%**
**>85%**	13.3%*	20.0%**	40.0%***	26.7%**

*0–14.9%

**15–29.9%

***30–44.9%

HCE, healthcare expenditure; IVD, *in vitro* diagnostic.

Physicians’ Expectations of IVD Markers. Regarding the prospective development of new IVD markers, 53% of physicians believed that IVD tests would need to demonstrate additional clinical evidence of improved patient outcomes (p<0.0001 vs other criteria), 29% stated that IVDs must provide health economic benefits plus evidence for improved patient outcome, whereas only 8% of physicians selected health economic benefits to be the exclusive purchasing factor. Overall, a significant proportion of physicians (83%) asked either for additional clinical data or combined clinical and health economic data. Thus, these combined health economic and outcomes benefits were more frequently requested than the sole provision of a diagnosis (p<0.0001), indicating that the latter will be insufficient to cater for the future demand of physicians.

## Discussion

To our knowledge this is the first comprehensive analysis to investigate the relationship between the value of IVDs and their associated cost in two major developed markets. Such an analysis is particularly important as recognition and reimbursement levels for IVDs have decreased significantly within the last 15 years [32]. Assessing the IVD utility–cost ratio is therefore important in raising awareness of IVDs as a cost-efficient tool for patient management. The present study has revealed at least four important findings:

1. The actual IVD spend as a part of overall HCE is low compared with other segments of the health value chain, accounting for 2.3% and 1.4% in the US and Germany, respectively.
2. IVD testing guides approximately 66% of clinical decisions.
3. Physicians overrate the costs of IVD as a proportion of HCE.
4. Physicians demand diagnostic tests that show both clinical utility and cost-effectiveness.

1. Our investigations revealed that the HCE on IVD in the US and Germany is 2.3% and 1.4%. This is in line with the statement from the Lewin Group report, concluding that diagnostics account for <5% of hospital costs and about 1.6% of all Medicare costs [26]. This cost is rather low when compared with other segments of the medical value chain, such as pharmaceuticals and medical aids, which in Germany accounted for 15% and 5%, respectively, of public HCE in 2013 [29]. Pharmaceutical spending on prescription medicines and over-the-counter products as a proportion of the overall HCE in 2013 was estimated to be 11.9% in the US and 17.5% in Canada [33].

As the percentage spent on IVDs relative to total HCE has remained fairly stable over the last 20 years, the results of the present study indicate that IVDs have contributed to the growth of the HCE at a constant low level (Fig 2). However, despite continuous discussion about cost containment, it must not be forgotten that newer predictive companion diagnostics are economically favorable. They allow patients who will benefit from a specific treatment to be identified and treated, while those who will not respond do not incur the cost for ineffective treatment and management of possible side effects. Indeed, evidence exists for a high cost–benefit ratio for identifying patients with KRAS and BRAF wild-type metastatic colorectal cancer suitable for treatment with cetuximab and those with HER2-positive breast carcinomas who will respond to trastuzumab [34, 35]. Furthermore, IVD-based screening programs may allow a reduction in the number of expensive late-stage treatments through earlier interventions [8].

2. The present study confirms the widespread belief that IVDs play an important role in clinical practice, as they influence 66% of clinical decision-making. This verifies the statement from the Lewin Group, which reported this number to be between 60–70% [26], which was a central aim of our study. Our investigation shows that clinical chemistry and hematology assessments play a pivotal role for clinical decision making in the initial patient work-up phase. This holds true for both the cardiology and oncology disease areas. Major differences in the use of molecular testing between the oncology and cardiology settings illustrates the excellent progress made in the field of personalized healthcare in cancer management but concerns remain over the low use of molecular testing in the cardiology field. Not surprisingly caution regarding the future of hypertension pharmacogenetics is warranted in various studies [36].

The strong influence of IVD on clinical decisions also underlines the responsibility of diagnostic laboratories and companies to physicians and patients. The manufacturers of IVD products play an important role in the reduction of laboratory errors by ensuring the highest possible safety and efficacy of their products [37]. Despite that fact that pre-analytical and post-analytical steps are more error prone than the analytical phase and errors due to analytical problems have been significantly reduced over the last two decades, laboratory errors are known to have a serious impact on patients and their safety [38,39].

While there has been substantial progress in reducing errors associated with IVD testing, additional challenges in the reduction of diagnostic errors and hence patient safety remain to be addressed [40,41]. In fact, the frequency of diagnostic errors related to IVD may still be as high as one out of 330 tests [38]. A recent publication from the US Institute of Medicine addresses this challenge with eight goals for improving diagnosis [40].

**Fig 3:**
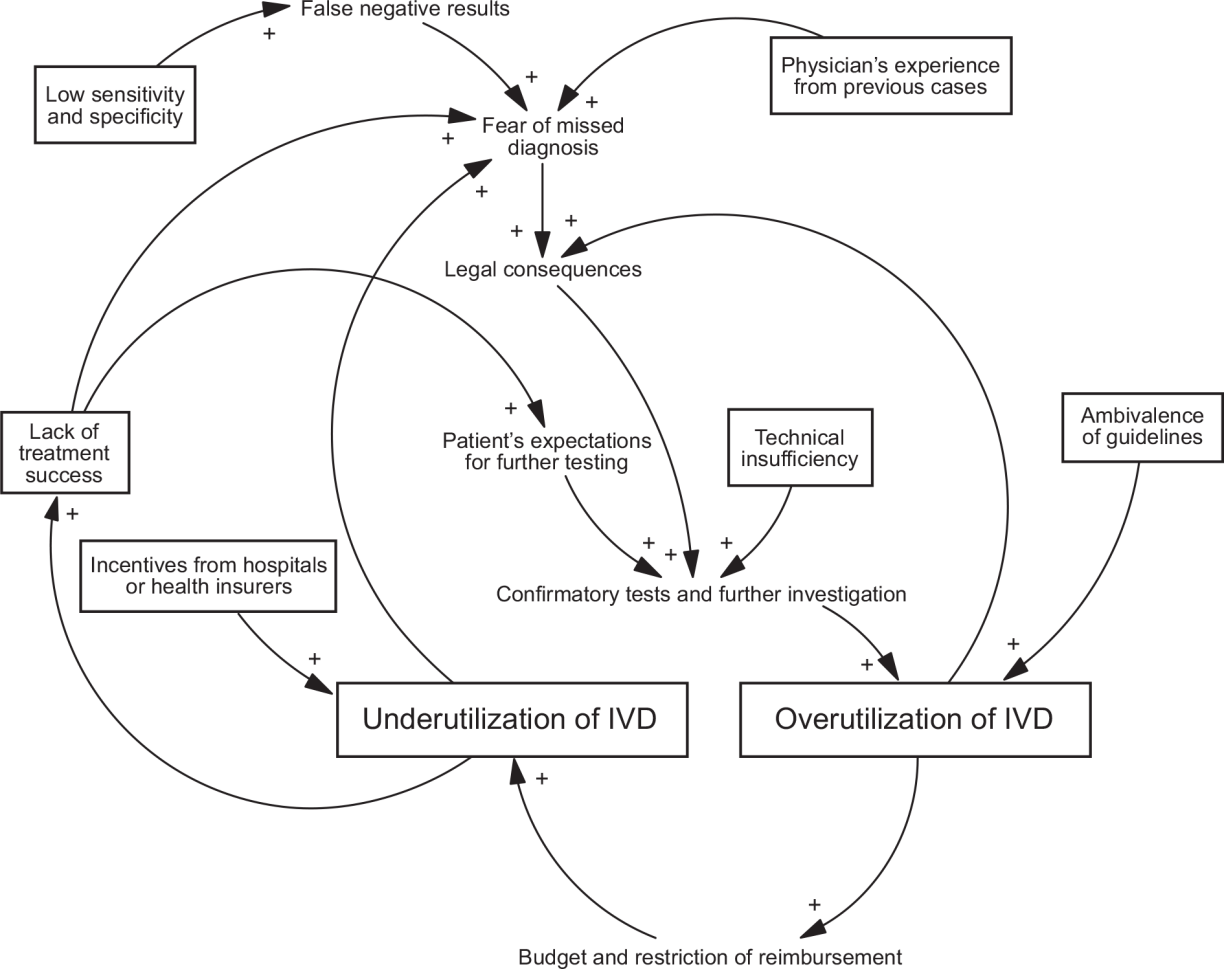
Causal loop diagram displaying the root causes for over- and under-utilization of IVD testing. Key drivers displayed in boxes; antecedents and secondary drivers displayed as plain text. IVD, *in vitro* diagnostic; →(+), positive causal links amplifying the behavior of target variable.

3. In the present study, IVD costs were generally overestimated by physicians. This might be triggered by the general belief of IVD overutilization, which has reported to be between 10–50% [42]. However, a recent meta-analysis suggests that IVD underutilization is more prevalent than overutilization (44.8% vs. 20.6%) [43]. Interestingly is also the fact that there were four times as many studies found on over- compared to underutilization during the assessed 15-year period and, despite in-depth literature research, only 42 studies finally matched the criteria for review, indicating a poor level of evidence on inappropriate use of IVD testing [43]. Root causes for IVD over- and underutilization are summarized in the causal-loop diagram shown in Fig 3.

Recently, the American Board of Internal Medicine has launched the “Choosing Wisely” initiative [44], which aims to identify tests of little clinical value with potential for over-diagnosis [24]. The Swiss Society of Internal Medicine launched a similar campaign in 2014 called “Smarter Medicine” [45].

Despite initial overestimation of IVD expenditure in the present analysis, most physicians rated the actual IVD cost as appropriate or too low, clearly demonstrating a low awareness of price structures among healthcare professionals. In a French study investigating cost awareness of overall hospital expenditure among physicians, only 29% of their overall ratings were within 50% of the true costs [46]. In another survey, only 19% of general practitioners estimated the true costs of laboratory and radiology tests in hospitals to be within 25% of the actual range [47]. This is in line with the present study results, which indicate that only 19% of physicians surveyed correctly estimated actual IVD costs.

4. The last finding of this analysis is that the mere supply of diagnostic tests will not be sufficient for physicians in the future, because evidence of accuracy does not automatically transfer to evidence of efficiency [48]. The present study has shown that >50% of all physicians demand proven clinical utility. These results reflect those of a study in which service provision (defined as the provision of validated treatment algorithms) was rated as a significantly stronger purchasing factor than technical preciseness [49]. There is evidence to suggest that physicians are reluctant to use diagnostic tools when a test result cannot be sufficiently translated into clinical actions [50]. Medical value in terms of clinical utility studies is able to close this gap by demonstrating improved patient outcomes by either decreasing triage time [51] or appropriate choice of treatment (companion diagnostics) [52].

The results of the present study furthermore demonstrate that 29% of all physicians demand new IVD markers with health economic benefits. Although pharmaceutical companies incorporated the concept of economic value decades ago, vendors of IVDs still see themselves primarily as providers of accurate technical equipment. This results in limited awareness of the economic value of IVDs, neglecting the fact that regular testing can fundamentally reduce healthcare costs, especially over the long term. In the US alone, US$1.3 billion could have been saved in 2004 if half of the patients with atrial fibrillation in routine medical care were optimally treated with oral anticoagulation [53].

### Limitations

This analysis has several limitations. The literature review was based on available public sources. As a result of a lack of some reference points, calculations and assumptions were necessary to fill gaps, which can lead to deviations from the actual spending. In Germany, the IVD cost as a percentage of HCE was calculated based on public HCE only, whereas total HCE was assessed in the US. An additional problem was the absence of a clear definition of “healthcare spending”. Consequently, associated costs may differ between the US and Germany. The survey included a relatively small sample size of interviewed physicians and there is a need for validation of the result using a larger sample base. In addition, the study was conducted for two developed markets only and thus validity of the results for the rest of the world remains to be proven.

### Conclusions

IVDs are an indispensable tool in clinical practice as they govern approximately 66% of clinical decision-making while accounting for approximately 2.3% and 1.4% of healthcare spending in the US and Germany, respectively. Although the presumed HCE on IVDs is generally overestimated by the majority of physicians, actual costs were considered as appropriate. IVDs can be regarded a cost-effective measure to maximize treatment outcomes. When used with established diagnostic algorithms, IVD testing can reduce direct and indirect healthcare costs [24], generate better clinical outcomes [54], and thus create Medical Value [49].

## Supporting Information

S1 Fig Average (cardiologists, oncologists, USA, Germany) percentage of IVD subtype use and average rated importance of IVD subtype use during initial patient workup. (DOCX)

S1 File Stage 2 –Oncology questionnaire. (DOCX)

S2 File Stage 2 –Cardiology questionnaire. (DOCX)

S3 File Stage 2 –Interview answers from oncologists and cardiologists. (XLSX)

S4 File Stage 3 –SERMO questions. (DOCX)

S5 File Stage 3 –Physician answers to SERMO questions. (XLSX)

S1 Table IVD Total Spending and Percentage of HCE 1993–2013 for US and Germany. (DOCX)

S2 Table IVD subtype usage and rated importance. A) Percentage of IVD subtype use during initial patient workup according to specialty and country; B) Rated importance of IVD subtypes for clinical practice and decision making during initial patient workup according to specialty and country (rating based on Likert scale, 1 = very low, 5 very high).
(DOCX)
