# Correction: The Value of *In Vitro* Diagnostic Testing in Medical Practice: A Status Report

**DOI:** 10.1371/journal.pone.0154008

**Published:** 2016-04-14

**Authors:** The PLOS ONE Staff

In the Competing Interests statement, the inclusion of Carmen Binder as a Roche employee is incorrect. The publisher apologizes for the error. The correct Competing Interests statement is as follows: U-PR, HHS and CGMM are employees of F. Hoffmann-La Roche Ltd. None of the other authors have any competing interests to declare. This does not alter our adherence to PLOS ONE policies on sharing data and materials.

There are errors in the Author Contributions. The publisher apologizes for the error. The correct contributions are: Conceived and designed the experiments: U-PR, TD, HHS. Performed the experiments: HHS, TD. Analyzed the data: CB, HHS, FG. Contributed reagents/materials/analysis tools: FG, CGMM, CB. Wrote the manuscript: U-PR, HHS, CB, ET, HM.
