# Supplementary material for: The Value of In Vitro Diagnostic Testing in Medical Practice: A Status Report
Source: PLoS One. 2016 Mar 4;11(3):e0149856. doi: 10.1371/journal.pone.0149856 (PMC4778800; doi:10.1371/journal.pone.0149856)
Supplement: S1 Fig — (DOCX) [file pone.0149856.s001.docx]

**S1 Fig:** Average (cardiologists, oncologists, USA, Germany) percentage of IVD subtype use and average rated importance of IVD subtype use during initial patient workup


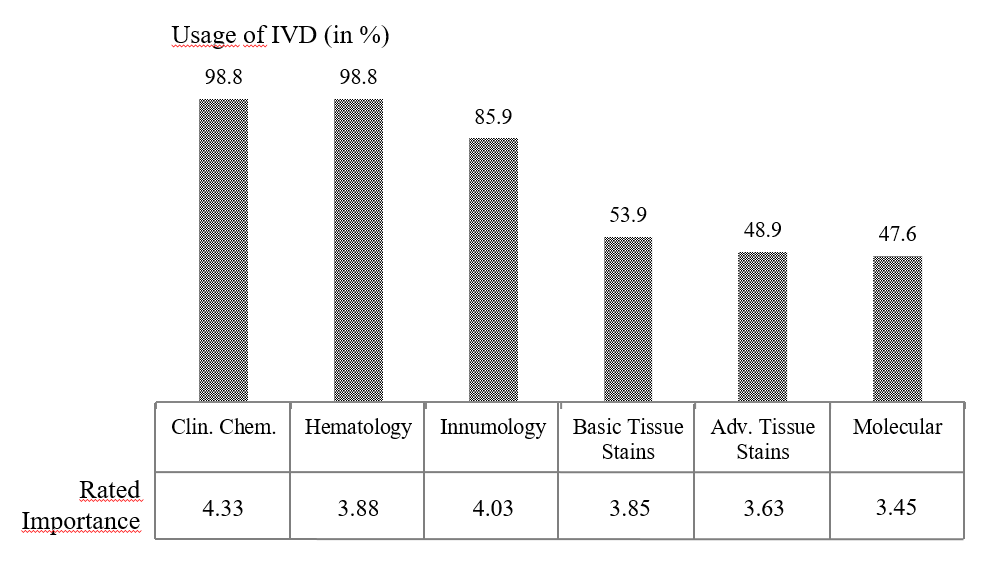


Rated according to Likert scale: 1=very low, 5 very high, Clin.Chem. Clinical chemistry, adv: advanced.
