# Supplementary material for: The Value of In Vitro Diagnostic Testing in Medical Practice: A Status Report
Source: PLoS One. 2016 Mar 4;11(3):e0149856. doi: 10.1371/journal.pone.0149856 (PMC4778800; doi:10.1371/journal.pone.0149856)
Supplement: S1 File — (DOCX) [file pone.0149856.s002.docx]

Date: _____ EAC ID: _______ Respondent ID: _______ By: __ Survey ID: ____

**Respondent**:

| **Name** | **Prefix** | **First name** | | **Last name** | | | | **Suffix** |  |
| --- | --- | --- | --- | --- | --- | --- | --- | --- | --- |
|  |  |  | |  | | | |  |  |
| **Title** |  | | | | | | | |  |
| **Department** |  | | | | | | | |  |
| **Phone** | **Direct** | | **Lab/Office** | | | | **Cell** | | |
|  |  | |  | | | |  | | |
| **e-mail** |  | | | | **Fax** |  | | | |

**Institution:**

| **Institute** |  | | | | |
| --- | --- | --- | --- | --- | --- |
| **Address1** | **Street** |  | | | |
| **Address2** | **Street/PO** |  | | | |
| **City/State** | **City** |  | | **State/Province** |  |
| **Country/Zip** | **Country** |  | | **Postal Code** |  |
| **Phone** |  | | **Metro Area** |  | |

**Institution type: a) Community Hospital b) Academic Center c) Cancer Center d) Private Doctor**

**Introduction**

*EAC is an international healthcare consulting and market research firm located in Stamford, CT. We are currently conducting an international study to understand the importance of clinical laboratory diagnostics in clinical decision making in the field of oncology,*

*Various published documents and reports in clinical medicine have claimed that laboratory diagnostics accounts for a mere 2% of healthcare spending but these tests are used in 70% of clinical decisions. We are seeking to better understand the impact that Molecular, Immuno, Tissue tests and other routine laboratory tests have on your clinical decision making and how it has changed from 10 years ago and how it may change in the future. In addition, the field of Personalized Medicine is of growing interest and has been the subject of numerous conferences, articles and symposia. We would like to understand the impact that Personalized Medicine is having in your oncology practice.*


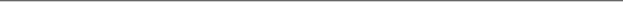


**Oncology Screener Questions (Ask during recruiting process)**

1. **Are you a Medical Oncologist (as opposed to surgical or radiation oncologist) Yes No**

**If No, STOP**

1. **Do you see at least 20 patients per week in your clinical practice? Yes No If No, STOP**
2. **Do you specialize on only 1 type of cancer? Yes No**

**If yes, which cancer do you specialize? _________________**

**ONCOLOGY INTERVIEW GUIDE**

**Background**

1. **How many patients do you typically see each week? _____________**
2. **Thinking about a typical week, what percentage of patients are:**
   1. ***New* patients (-Patients for whom you are in the process of confirming a diagnosis and or establishing an initial treatment plan) ________%**
   2. **Patients currently undergoing treatment regimens ______%**
   3. **Patients in post-treatment follow-up phase __________%**

**(note: a breast cancer patient on long term 5 year tamoxifen therapy or similar would be considered post-treatment category)**

1. **Thinking about the all the patients you see in a typical week, what percentage of patient visits do you order any laboratory diagnostic test? (This can be a CBC, chemistry panel, urinalysis, tissue test, immunology, molecular test or any other lab test. We are not including EKG, imaging or any other type of *in-vivo* test) ________%**
   1. **Now, in those patients where a laboratory test of some kind is ordered, what percentage of your clinical decisions related to the starting, changing or stopping of a particular therapy are impacted by a laboratory test? For this question, we are excluding in vivo imaging, EKGs, etc. _______%**

**(Be sure they understand differentce between Q3 and 3a)**

**Questions about Initial Diagnosis (note: now we are back to talking about ALL lab tests)**

1. **Thinking about patients in the initial diagnosis phase (including those just referred to you who have been diagnosed by another physician), in what percentage do you order laboratory diagnostic tests in this initial work-up phase? (Include routine tests, immunology, flow cytometry, anatomic pathology, Molecular/DNA tests, etc.) ________%**
2. **For each of the following tests that might be included in an initial patient work up, please indicate whether you regularly order the test and the relative importance of the test in your decision making (Score on 1 to 5 scale with 5 being highest importance).**

**(Note: we are looking for relative importance of each type of test in the initial work up. I would expect molecular and tissue stains will be very important here while routine chem is much less)**

| **Test type** | **Ordered With Initial Consult**  **Yes/No** | Comments    **Importance in Clinical Decisions (1-5 scale)** |
| --- | --- | --- |
| **Routine Chemistry** |  |  |
| **Routine Hematology** |  |  |
| **Flow cytometry** |  |  |
| **Serum tumor markers/ Other immunology** |  |  |
| **Basic Tissue Stains (H&E and Special)** |  |  |
| **Advanced Tissue Stains**  **(IHC & FISH)** |  |  |
| **Molecular/DNA/genomic tests** |  |  |

**Patients Undergoing Treatment**

1. **Thinking about all of your patients who are currently undergoing treatment for a cancer, what percentage do you order any laboratory diagnostic test at each visit? (or a pre-visit order). _________% (example: white cell counts, liver function, metabolic panel, flow cytometry, serum tumor marker)**

1. **For each of the following tests that might be included in an on-going treatment plan, please indicate whether you regularly order the test and the relative importance of the test in your decision making (Score on 1 to 5 scale with 5 being highest importance).**

| **Test type** | **Ordered With On-going treatment**  **Yes/No** | Comments  **Importance in Clinical Decisions (1-5 Scale)** |
| --- | --- | --- |
| **Routine Chemistry** |  |  |
| **Routine Hematology** |  |  |
| **Flow cytometry** |  |  |
| **Serum tumor markers/ Other immunology** |  |  |
| **Basic Tissue Stains (H&E and Special)** |  |  |
| **Advanced Tissue Stains**  **(IHC & FISH)** |  |  |
| **Molecular/DNA/genomic tests** |  |  |

**Post Treatment Follow-Up**

1. **Thinking about patients who are currently in the post-treatment follow up phase, in what percentage do you order laboratory diagnostic tests at each visit? (or a pre-visit order). _______% (this could be a CBC, chemistry panel, immunology serum marker like PSA, etc.)**

**(Note: some cancers may rely much more on scans versus lab tests in follow up)**

1. **For each of the following tests that might be included in post- treatment follow up plan, please indicate whether you regularly order the test and the relative importance of the test in your decision making (Score on 1 to 5 scale with 5 being highest importance).**

| **Test type** | **Ordered With Post treatment follow up**  **Yes/No** | Comments  **Importance in Clinical Decisions (1-5 Scale)** |
| --- | --- | --- |
| **Routine Chemistry** |  |  |
| **Routine Hematology** |  |  |
| **Flow cytometry** |  |  |
| **Serum tumor markers/ Other immunology** |  |  |
| **Basic Tissue Stains (H&E and Special)** |  |  |
| **Advanced Tissue Stains**  **(IHC & FISH)** |  |  |
| **Molecular/DNA tests** |  |  |

***Thank you for your participation today!***

**Honorarium Information:**

**Amount: ____$250___________**

**US** 🞎 Amazon.com 🞎 iTunes 🞎 Declined

**Email for e-gift card: ______________________________________________________________**

**OR**

**2. Send Check to:x🞎 Home 🞎 Work 🞎 Donation to major charity**

Name on Check: ______

**Mailing Address:**

Institution: _____________________________________________________________

Department ____________________________________________________________

Street _________________

Address2: ____________________________________________________________

City, State, Province _____________________

Zip Code /Postal code___________

Country: ______________________
