# Supplementary material for: The Value of In Vitro Diagnostic Testing in Medical Practice: A Status Report
Source: PLoS One. 2016 Mar 4;11(3):e0149856. doi: 10.1371/journal.pone.0149856 (PMC4778800; doi:10.1371/journal.pone.0149856)
Supplement: S2 File — (DOCX) [file pone.0149856.s003.docx]

Date: __________ EAC ID: _______ Respondent ID: _______ By: ______ Survey ID: ______

**Respondent**:

| **Name** | **Prefix** | **First name** | | **Last name** | | | | **Suffix** |  |
| --- | --- | --- | --- | --- | --- | --- | --- | --- | --- |
|  |  |  | |  | | | |  |  |
| **Title** |  | | | | | | | |  |
| **Department** |  | | | | | | | |  |
| **Phone** | **Direct** | | **Lab/Office** | | | | **Cell** | | |
|  |  | |  | | | |  | | |
| **e-mail** |  | | | | **Fax** |  | | | |

**Institution:**

| **Institute** |  | | | | |
| --- | --- | --- | --- | --- | --- |
| **Address1** | **Street** |  | | | |
| **Address2** | **Street/PO** |  | | | |
| **City/State** | **City** |  | | **State/Province** |  |
| **Country/Zip** | **Country** |  | | **Postal Code** |  |
| **Phone** |  | | **Metro Area** |  | |

**Institution type: a) Community Hospital b) Academic Center c) Private Doctor**

**Introduction**

*EAC is an international healthcare consulting and market research firm located in Stamford, CT. We are currently conducting an international market study to understand the importance of clinical laboratory diagnostics in clinical decision making in the field of cardiology*

*Various published documents and reports in clinical medicine have claimed that laboratory diagnostics accounts for a mere 2% of healthcare spending but these tests are used in 70% of clinical decisions. We are seeking to better understand the impact that laboratory tests have on your clinical decision making and how it has changed from 10 years ago and how it may change in the future. Also, the use of genetic tests for genotyping or so-called “personalized medicine” is gaining attention. We would like to see if these types of tests are gaining adoption in the field of cardiology*


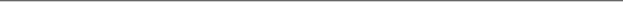


**Cardiology Screener Questions (Ask during recruiting process)**

1. **Are you a general cardiologist or an interventional cardiologist? General Interventional**

1. **Do you see at least 20 patients per week in your clinical practice?**

**Yes No If No, STOP**

1. **Do you specialize on only specific disease area in cardiology? Yes No**
   1. **If yes, which area_______________**

**CARDIOLOGY INTERVIEW GUIDE**

**Background**

1. **How many patients do you typically see each week? _____________**
2. **What percentage are *new* patients (first time visit)? __________**
3. **Thinking about the patients you see in a typical week, what percentage of patient visits do you order a laboratory diagnostic blood, urine or tissue test? (This can be a CBC, chemistry panel, urinalysis, tissue test, cardiac markers for troponin, BNP, hs-CRP, lipid panel, INR, molecular test or any other lab test. We are not including EKG, stress tests, blood pressure, nuclear imaging, ultrasound or any other type of *in-vivo* test) ________%**

**a.Now, in those patients where a laboratory test of some kind is ordered, what percentage of your clinical decisions related to the starting, changing or stopping of a particular therapy are impacted by a laboratory test? For this question, we are excluding in vivo imaging, EKGs, etc. ________%**

**(Be sure they understand and differentiate between Q3 and 3a)**

**Questions about Initial Diagnosis (note: now we are back to talking about ALL lab tests)**

1. **Thinking about patients in the initial diagnosis phase (including those just referred to you who have been diagnosed by another physician), in what percentage do you order laboratory diagnostic tests in this initial work-up phase? (Include routine chemistry, hematology tests, immunology, anatomic pathology, Molecular/DNA tests, etc.) _______%
   (note: just remind physician that this question is for initial diagnosis, unlike Q3)**
2. **For each of the following tests that might be included in an initial patient work up, please indicate whether you regularly order the test and the relative importance of the test in your decision making (Score on 1 to 5 scale with 5 being highest importance).**

| **Test type** | **Ordered With Initial Consult**  **Yes/No** | Commentsents:  **Importance in Clinical Decisions (1-5 scale)** |
| --- | --- | --- |
| **Routine Chemistry (e.g. lipids, electrolytes)** |  |  |
| **Routine Hematology** |  |  |
| **Coagulation (INR, other coagulation tests)** |  |  |
| **Immunology tests (e.g. cardiac markers, BNP)** |  |  |
| **Basic Tissue Stains (H&E and Special)** |  |  |
| **Advanced Tissue Stains**  **(IHC & FISH)** |  |  |
| **Molecular/DNA/genomic tests** |  |  |

**Perhaps some qualitative comments here on what is ordered and why**

**Comments:**

**Patients Undergoing Treatment and Follow-up**

1. **Thinking about all of your patients who are currently undergoing treatment for a cardiovascular issue, what percentage do you order any laboratory diagnostic test at each visit? (or a pre-visit order). _________% (example: blood counts, lipids, chemistry profile, PT/INR) (Example: Patients on statin drugs might be lipid tests, Warfarin patients get regular INRs)**
2. **For each of the following tests that might be included in an on-going treatment plan or follow up plan, please indicate whether you regularly order the test and the relative importance of the test in your decision making (Score on 1 to 5 scale with 5 being highest importance).**

| **Test type** | **Ordered With On-going treatment**  **Yes/No** | Commentsents:  **Importance in Clinical Decisions (1-5 Scale)** |
| --- | --- | --- |
| **Routine Chemistry** |  |  |
| **Routine Hematology** |  |  |
| **Coagulation tests** |  |  |
| **Immunology tests (e.g. cardiac markers, BNP)** |  |  |
| **Basic Tissue Stains (H&E and Special)** |  |  |
| **Advanced Tissue Stains**  **(IHC & FISH)** |  |  |
| **Molecular/DNA/genomic tests** |  |  |

***Thank you for your participation today!***

**Honorarium Information: (Not applicable for Germany)**

**Amount: _______________**

**2. Send Check to:🞎 Home 🞎 Work 🞎 Donation to major charity**

Name on Check: ________________________________

**Mailing Address:**

Institution: _____________________________________________________________

Department ____________________________________________________________

Street ______________________________________________________________

Address2: ____________________________________________________________

City, State, Province ______________________________________________________

Zip Code /Postal code___________________________________
