# Supplementary material for: The Value of In Vitro Diagnostic Testing in Medical Practice: A Status Report
Source: PLoS One. 2016 Mar 4;11(3):e0149856. doi: 10.1371/journal.pone.0149856 (PMC4778800; doi:10.1371/journal.pone.0149856)
Supplement: S4 File — (DOCX) [file pone.0149856.s005.docx]

Stage 3 SERMO Questions

PULSE Preview

Value of Diagnostics

1) What do you think, is the average cost impact of in vitro diagnostic testing (including all molecular assays, protein assays, tissue based assays) on your national health care spending? (select one answer)

*Choose one answer:*

- 0-4%FOLLOW UP
- 5-10% FOLLOW UP
- 11-20% FOLLOW UP
- >20% FOLLOW UP

2) A literature review yielded that actually diagnostic spent is less than 2% of the overall health care (HC) costs. Do you think this is appropriate? (Select one answer)

*Choose one answer:*

- It is too low; Innovative assays deserve a higher price even if total health care spent increases
- It is too low; Innovative assays deserve a higher price but cuts must be made in other HC segments
- It is about right and should be continued over time on that level
- It is too high. It should be decreased and added to other segments to the HC value chain.
- It is too high. It should be decreased to reduce the overall HC spent.

3) What do you think is most relevant for a new Assay in order to be used by you? (One answer that resembles best your thinking)

*Choose one answer:*

- I request/establish new markers in my lab due to costs and time constraints
- Assays must only be available; A “research use only” assay is good enough
- Assays must be available and show clinical evidence for improved patient outcome
- Assays must be available and show health economic benefits (cost reduction)
- Availability, clinical evidence for improved patient outcome, and health economic benefits
