# Supplementary material for: The Value of In Vitro Diagnostic Testing in Medical Practice: A Status Report
Source: PLoS One. 2016 Mar 4;11(3):e0149856. doi: 10.1371/journal.pone.0149856 (PMC4778800; doi:10.1371/journal.pone.0149856)
Supplement: S1 Table — (DOCX) [file pone.0149856.s007.docx]

**S1 Table:** IVD Total Spending and Percentage of HCE 1993-2013 for US and Germany

|  | United States | | | Germany | | |
| --- | --- | --- | --- | --- | --- | --- |
| Year | Total IVD spending ($ M) | Annual growth of IVD spending | IVD of total HCE^a^ | Government IVD spending (€ M) | Annual growth of government IVD spending | IVD of total government HCE^a^ |
| 1993 | 24,000^b^ | - | 2.6% | 2,389^f^ | - | 1.5% |
| 1994 | 25,281^a^ | 5.3%^c^ | 2.6% | 2,514^f^ | 5.5%^a^ | 1.4% |
| 1995 | 25,265^a^ | 5.3%^c^ | 2.6% | 2,623^f^ | 4.3%^a^ | 1.4% |
| 1996 | 26,596^a^ | 5.3%^c^ | 2.6% | 2,641^f^ | 0.7%^a^ | 1.4% |
| 1997 | 27,998^a^ | 5.3%^c^ | 2.6% | 2,705^f^ | 2.4%^a^ | 1.4% |
| 1998 | 30,600^d^ | 3.8%^a^ | 2.5% | 2,777^f^ | 2.7%^a^ | 1.4% |
| 1999 | 30,700^d^ | 0.3%^a^ | 2.4% | 2,733^f^ | -1.6%^a^ | 1.3% |
| 2000 | 32,900^d^ | 7.2%^a^ | 2.4% | 2,850^f^ | 4.3%^a^ | 1.3% |
| 2001 | 35,400^d^ | 7.6%^a^ | 2.4% | 3,015^f^ | 5.8%^a^ | 1.4% |
| 2002 | 38,300^d^ | 8.2%^a^ | 2.3% | 3,149^f^ | 4.4%^a^ | 1.4% |
| 2003 | 40,100^d^ | 4.7%^a^ | 2.3% | 3,240^f^ | 2.9%^a^ | 1.4% |
| 2004 | 42,700^d^ | 6.5%^a^ | 2.2% | 3,265^f^ | 0.8%^a^ | 1.4% |
| 2005 | 45,400^d^ | 6.3%^a^ | 2.2% | 3,327^f^ | 1.9%^a^ | 1.4% |
| 2006 | 48,400^d^ | 6.6%^a^ | 2.2% | 3,441^f^ | 3.4%^a^ | 1.4% |
| 2007 | 51,600^d^ | 6.6%^a^ | 2.2% | 3,486^f^ | 1.3%^a^ | 1.4% |
| 2008 | 55,100^d,g^ | 6.8%^a^ | 2.3%^a,h^ | 3,825^f^ | 9.7%^a^ | 1.4% |
| 2009 | 57,304^a^ | 4.0%^e,i^ | 2.3% | 3,912^f^ | 2.3%^a^ | 1.4% |
| 2010 | 59,596^a^ | 4.0%^e^ | 2.3% | 3,984^h^ | 1.8%^a^ | 1.4% |
| 2011 | 61,980^a^ | 4.0%^e^ | 2.3% | 4,258^f^ | 6.9%^a^ | 1.4% |
| 2012 | 64,459^a^ | 4.0%^e^ | 2.3% | 4,201^f^ | -1.3%^a^ | 1.4% |
| 2013 | 67,038^a^ | 4.0%^e^ | 2.3% | 4,450^f^ | 5.9%^a^ | 1.4% |
| **Average** | | **5.3%^a^** | **2.3%** |  | **3.3%**^a^ | **1.4%** |

^a^Calculated values. ^b^Assumption for estimated IVD spending value in 1993. ^c^5.3% is the assumed growth rate for 1994-1997 based on overall 20-year average from 1993 to 2013. ^d^Total laboratory IVD spending: 1998–2008 [1]. ^e^4% growth rate assumption (2009–2013) [2]. ^f^All clinical laboratory spending figures : 1993–2011 [3]. ^g^Quest Diagnostics Investor Presentation, Barclays Capital 2011 Global Healthcare Conference, March 15, 2011 [4]. ^h^Calculated and also corroborated from [1]. ^i^Clinical Laboratory Services Market, 2010. Publisher: Kalorama, Inc (New York, NY) [4].

IVD: in-vitro diagnostics, HCE: health care expenditure, M: million, $: United States dollars, €: Euro

**References**

1. CLMA ThinkLab 2010. Laboratory Industry Outlook Report2010–2011. p3. Kalorama, Inc (New York, NY). Available at <http://c.ymcdn.com/sites/www.clma.org/resource/resmgr/Professional_Development_-_Past_ThinkLabs/305_Stephanie_Murg.pdf>
2. Quest Diagnostics Investor Presentation, JP Morgan Healthcare Conference, January 9, 2013). Available at http://ir.questdiagnostics.com/phoenix.zhtml?c=82068&p=irol-presentations
3. Health expenditures in Germany as share of GDP and in millions of Euro (absolute and per inhabitant). Available from: <http://www.gbe-bund.de/gbe10/trecherche.prc_them_rech?tk=19200&tk2=19300&p_uid=gast&p_aid=71688746&p_sprache=E&cnt_ut=10&ut=19310>
4. The Clinical Laboratory Services Market (Growth Opportunities, Competitive Analysis and Competitor Profiles). Available at [www.slideshare.net/MarketResearch.com/the-clinical-laboratory-services-market-growth-opportunities-competitive-analysis-and-competitor-profiles-7102180](http://www.slideshare.net/MarketResearch.com/the-clinical-laboratory-services-market-growth-opportunities-competitive-analysis-and-competitor-profiles-7102180)
