# Supplementary material for: The Value of In Vitro Diagnostic Testing in Medical Practice: A Status Report
Source: PLoS One. 2016 Mar 4;11(3):e0149856. doi: 10.1371/journal.pone.0149856 (PMC4778800; doi:10.1371/journal.pone.0149856)
Supplement: S2 Table — A) Percentage of IVD subtype use during initial patient workup according to specialty and country; B) Rated importance of IVD subtypes for clinical practice and decision making during initial patient workup according to specialty and country (rating based on Likert scale, 1 = very low, 5 very high). (DOCX) [file pone.0149856.s008.docx]

**S2 Table:** IVD subtype usage and rated importance

**A)**

| Specialty | Country | Clinical Chemistry | Hematology | Immunology | | Basic Tissue Stains | | Advanced Tissue Stains | | Molecular | |
| --- | --- | --- | --- | --- | --- | --- | --- | --- | --- | --- | --- |
| Oncology | USA | 95 | 95 | 75 | | 80 | | 95 | | 65 | |
|  | Germany | 100 | 100 | 100 | | 100 | | 75 | | 95 | |
|  | *Overall* | *97.5* | *97.5* | *57.5* | | *90* | | *85* | | *80* | |
| Cardiology | USA | 100 | 100 | 100 | | 15.7 | | 10.5 | | 10.5 | |
|  | Germany | 100 | 100 | 68.4 | | 20 | | 15 | | 20 | |
|  | *Overall* | *100* | *100* | *84.6* | | *17.9* | | *12.8* | | *15.3* | |
| **Overall** |  | **98.8** | **98.8** | **85.9** | | **53.9** | | **48.9** | | **47.6** | |
| **B)** |  |  |  |  |  | |  | |  | |  |
|  |  |  |  |  |  | |  | |  | |  |
| Specialty | Country | Clinical Chemistry | Hematology | Immunology | | Basic Tissue Stains | | Advanced Tissue Stains | | Molecular | |
| Oncology | USA | 4.2 | 4.1 | 3.7 | | 4.8 | | 4.6 | | 3.8 | |
|  | Germany | 4.1 | 4 | 3.8 | | 4.9 | | 4.8 | | 4.3 | |
|  | *Overall* | *4.14* | *4.05* | *3.75* | | *4.85* | | *4.7* | | *4.05* | |
| Cardiology | USA | 4.5 | 3.7 | 4.3 | | 2.4 | | 2.1 | | 2.5 | |
|  | Germany | 4.5 | 3.7 | 4.3 | | 3.3 | | 3 | | 3.2 | |
|  | *Overall* | *4.5* | *3.7* | *4.3* | | *2.58* | | *3.6* | | *2.85* | |
| **Overall** |  | **4.33** | **3.88** | **4.03** | | **3.85** | | **3.63** | | **3.45** | |

Table A: Percentage of IVD subtype use during initial patient workup according to specialty and country.

Table B: Rated importance of IVD subtypes for clinical practice and decision making during initial patient workup according to specialty and country (rating based on Likert scale, 1=very low, 5 very high).
